# Recent Advances in Organocatalytic Kinetic Resolution for the Synthesis of Axially Chiral Compounds

**DOI:** 10.3390/molecules31050786

**Published:** 2026-02-26

**Authors:** Liying Cui, Yin Zheng

**Affiliations:** 1 School of Chemistry and Chemical Engineering, North University of China, Taiyuan 030051, China; 2 College of Science, Nanjing Forestry University, Nanjing 210037, China; 3 National Key Laboratory for the Development and Utilization of Forest Food Resources, College of Chemical Engineering, Nanjing Forestry University, Nanjing 210037, China

**Keywords:** axially chiral compounds, asymmetric catalysis, kinetic resolution, non-covalent interactions

## Abstract

Axially chiral compounds, indispensable in asymmetric catalysis, drug discovery, and materials science, have witnessed transformative advancements in synthesis through organocatalytic kinetic resolution (OKR) over the past decade. This review systematically dissects the latest achievements (2010–2025) in OKR, focusing on catalyst design, mechanistic insights, substrate diversification, and synthetic applications across C–C biaryl, C–N heterobiaryl, and olefinic axially chiral frameworks. By harnessing non-covalent interactions, OKR has emerged as a powerful strategy to overcome the challenges of low rotational barriers and limited stereocontrol, offering sustainable and enantioselective access to privileged chiral scaffolds. Furthermore, the current challenges and future prospects in this rapidly evolving field are assessed.

## 1. Introduction

**Scheme 1:**
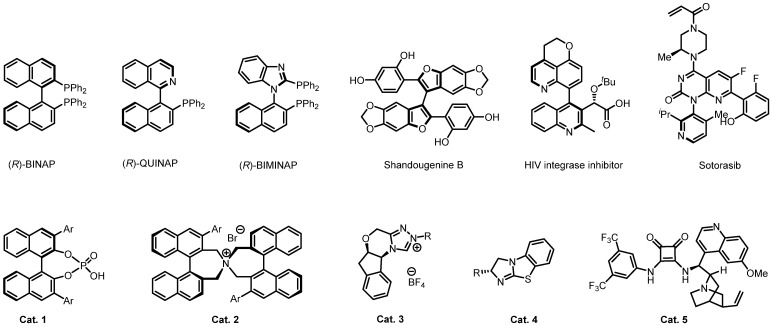
Representative axially chiral compounds and organocatalysts.

Axially chiral compounds constitute a pivotal class of molecular scaffolds, playing an indispensable role in modern asymmetric catalysis [1,2,3,4,5,6], organic synthesis [7,8,9] and medicinal chemistry [10,11,12,13,14,15]. Axially chiral compounds also appear in the field of materials science, especially in the synthesis of macrocycles. For example, the following have been recently reported: the first direct enantioselective synthesis of a mechanically axially chiral rotaxane, the synthesis of the binaphthyl-bithiophene macrocyclic dimer and trimer via a Ni(0)-mediated homocoupling reaction, the enantioselective synthesis of inherently chiral calix [4] arenes via chiral phosphoric acid (CPA)-catalyzed lower-rim asymmetric functionalization, a novel BINOL-embedded atropisomeric chiral oxa-nanographene obtained through the selective dimerization of azopine-containing, and hexa-peri-hexabenzocoronene-like aromatics. These axially chiral molecules are of great significance for developing functionalized compounds based on macrocyclics and have potential in optoelectronic materials and related fields [16,17,18,19]. Characterized by their distinctive rigid structures, axially chiral compounds effectively impede intramolecular rotation, enabling a precise control over spatial configurations, which has propelled them to the forefront of research interest. Classic examples, such as BINAP and its derivatives, have been extensively employed as chiral ligands or catalysts in asymmetric catalytic reactions, significantly enhancing both enantioselectivity and catalytic activity (Scheme 1). Moreover, axial chirality also constitutes a unique chiral element found in a wealth of naturally occurring bioactive compounds, such as Shandougenine B [20]. Over the past few decades, the efficient synthesis of axially chiral compounds has been a research topic of great interest [21,22,23,24,25,26,27,28,29].

Organocatalysis has become an indispensable component of asymmetric synthesis, providing access to previously elusive synthetic routes and enabling the efficient construction of complex chiral molecules vital to pharmaceutical, agrochemical, and materials science applications [30,31,32,33,34]. This transformative potential has been realized through the development of diverse organocatalytic systems, such as chiral phosphoric acids, quaternary ammonium salts, bifunctional hydrogen-bonding catalysts, *N*-heterocyclic carbenes, and isothiourea derivatives, each employing distinct activation modes to achieve precise stereocontrol.

A particularly compelling demonstration of organocatalysis lies in its application to construct axially chiral compounds [35,36,37,38]. The integration of organocatalysis and kinetic resolution (KR) protocols has established a robust platform for assembling functional chiral architectures, showing an exceptional utility in constructing challenging axially chiral scaffolds where exacting control over molecular conformation is paramount.

**Scheme 2:**
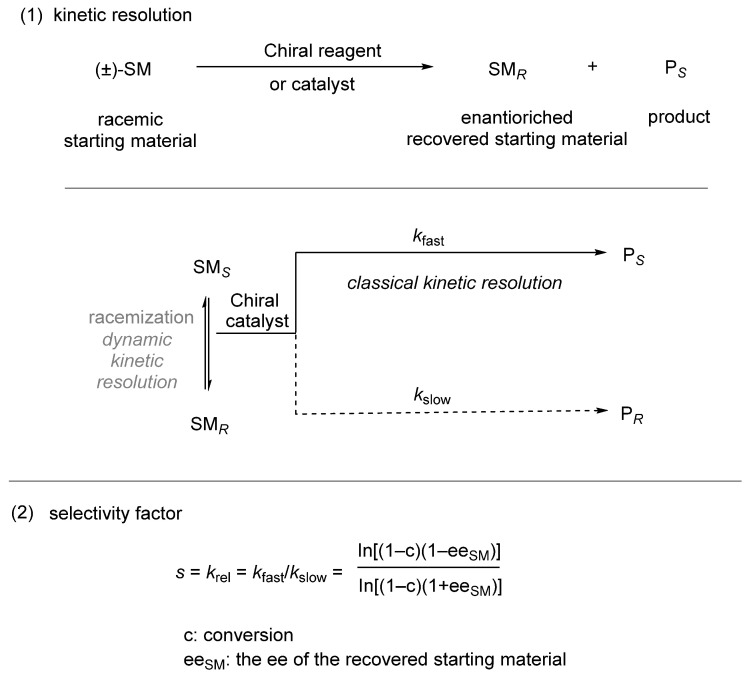
Kinetic resolution and the selectivity factor.

In the classical kinetic resolution, the racemic substrate ((±)-SM) undergoes a reaction in the presence of a chiral reagent or catalyst. The SM activated by the chiral compound generates two diastereomeric transition states, effectively lowering the activation energy of one enantiomer. If the reaction rate difference is sufficiently large, the rapidly reacting enantiomer will further convert to the corresponding chiral product (P*_S_*), while the slowly reacting enantiomer can be recovered unchanged (SM*_R_*) (Scheme 2). Since the proportion of each enantiomer is only 50% at the beginning of the reaction, the theoretical maximum yield of SM or P is 50%. Alternatively, when the starting substrate undergoes rapid racemization during the reaction, causing the two enantiomers to convert into the single enantiomer of the product, dynamic kinetic resolution is possible [39]. According to the Curtin–Hammett principle, a theoretical maximum yield can reach 100%. To fully utilize the advantages of dynamic kinetic resolution (DKR), some special requirements must be met, such as the irreversibility of the resolution step and the prevention of racemization of the product under reaction conditions. Furthermore, the rate constant of the racemization process should be faster than the rate constant of the resolution step.

Over the progress of a kinetic resolution reaction, the enantiomeric purity of the recovered starting material gradually increases, while the enantiomeric purity of the product gradually decreases. The efficiency of a kinetic resolution is typically evaluated by the selectivity factor (*s*) or the relative reaction rate (*k*_rel_ = *k*_fast_/*k*_slow_, where *k*_fast_ and *k*_slow_ are the fast and slow reaction rates of the enantiomers, respectively), which reflects the energy difference between the two diastereomeric transition states in the selectivity-determining step of the catalytic reaction. A high selectivity factor is necessary for obtaining the high ee product in kinetic resolution. If the reaction is a first-order in substrate, the equation proposed by Kagan and Fiaud can be used, which correlates the conversion (c) and the enantiomeric excess of the recovered starting material (ee_SM_) with *k*_rel_ or the selectivity factor (*s*) [40].

This review summarizes recent progress in organocatalytic kinetic resolution (OKR) for constructing three major classes of axially chiral compounds, C-C biaryls, C-N heterobiaryls, and olefinic systems, highlighting mechanistic innovations and synthetic versatility.

## 2. Organocatalytic Kinetic Resolution of C-C Biaryl Axially Chiral Molecules

### 2.1. Organocatalytic Kinetic Resolution of Axially Chiral Biaryl Diols

Axially chiral biaryl diols (e.g., BINOL) represent a privileged scaffold in synthetic chemistry, enabling precise stereoinduction in a series of asymmetric transformations, rendering them indispensable as ligands, organocatalysts, and building blocks for chiral materials [41,42,43,44]. In this context, organocatalytic kinetic resolution via asymmetric protection emerges as a robust alternative to traditional stoichiometric resolution methods, effectively addressing the limited substrate scope inherent to catalytic methods, such as the oxidative coupling of naphthols, while enabling efficient and practical large-scale synthesis.

**Scheme 3:**
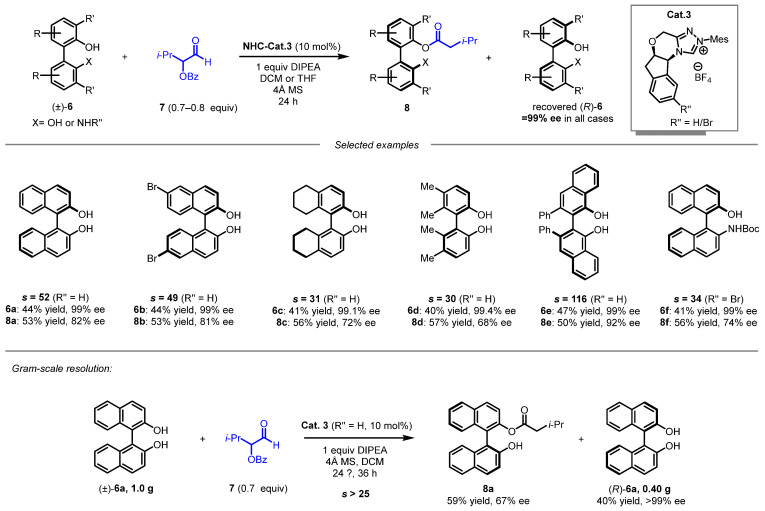
NHC-catalyzed atroposelective acylation enables kinetic resolution of 1,1′-biaryl-2,2′-diols.

Historically, asymmetric kinetic resolution (AKR) acylation reactions have been well established for resolving racemic alcohols into chiral counterparts [45]. Nevertheless, applying these methods to the synthesis of axially chiral biaryl alcohols has proven to be significantly challenging. A pivotal breakthrough was achieved in 2014 by Zhao and co-workers, who developed a highly efficient *N*-heterocyclic carbene (NHC)-catalyzed system for the enantioselective acylation of axially chiral alcohols (Scheme 3) [46]. In this approach, chiral NHCs Cat.**3**, generated in situ from azolium precursors, activated aldehydes to form chiral acyl azolium intermediates. This innovation facilitated the enantioselective acylation of racemic substrates **6** with remarkable efficacy.

The system demonstrated broad substrate compatibility, accommodating diverse substituted binaphthyls, biphenyls, non-*C*_2_-symmetric diols and *N*-Boc-protected NOBIN derivatives, while effectively circumventing the typical side reactions of unprotected amino alcohols. The results from this catalytic system were outstanding; for instance, recovered axially chiral diols **6a**–**e** and amino alcohols **6f** exhibited enantiomeric excess reaching 99% and selectivity factors (*s*-factors) up to 116. Notably, the protocol was highly scalable to gram quantities, allowing for the production of enantiopure BINOL (*R*)-**6a** (>99% ee) with a 40% yield, further underscoring its practicality for large-scale applications. However, biphenyl diols with 6,6′-position substituents exhibited relatively low selectivities; for instance, for such substrate bearing small methoxy groups, the *s* value in the reaction was only 22.

**Scheme 4:**
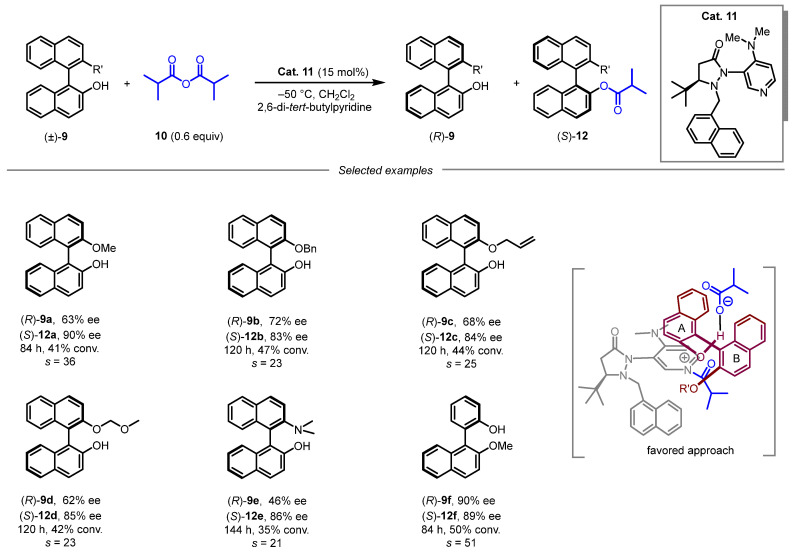
Chiral DMAP-catalyzed kinetic resolution of axially chiral biaryl diols.

Simultaneously, the Sibi group reported the utilization of a fluxionally chiral 4-dimethylaminopyridine (DMAP) catalyst for acylation kinetic resolution [47]. The results of catalyst screening demonstrated that DMAP-based Cat.**11** exhibited a good performance in the kinetic resolution of the racemic BINOL derivatives **9** (Scheme 4). By employing isobutyric anhydride as the acylation reagent alongside bulky 2,6-di-*tert*-butylpyridine as an additive base, the *s* factor achieved peaks of up to 51. This method was applicable to the kinetic resolution of various racemic secondary alcohols, 2-hydroxy-1,1′-biaryl analogs and a 2-amino-2′-hydroxy-1,1′-binaphthyl (nobin) derivative (**9e**). For the 2-naphthyl-substituted and propargylic secondary alcohols, they could be efficiently resolved within 48 h with *s* = 17–37, while the resolution selectivity for 1-naphthyl-substituted secondary alcohols was relatively lower (*s* = 6). Additionally, the larger steric hindrance of the alkyl groups in aliphatic secondary alcohols usually led to higher s factors. Another class of 2-hydroxy-1,1′-biaryl analogs also achieved good to excellent selectivity (*s* = 10–51) in this method. Notably, biaryl compounds without a substituent or with electron-deficient groups at the β’ position gave lower selectivities in this reaction.

The reported modular catalyst **11** consisted of a DMAP catalytic site, a chiral pyrazolidinone, and a tunable fluxional substituent (1-naphthalen-1-ylmethyl), where the latter enhanced stereoselectivity through steric hindrance control. Meanwhile, according to the X-ray single-crystal structural analysis of the catalyst by the authors, the fluxional group could shield specific faces of the pyridine ring, thereby directing the approach of the substrate. Their study confirmed that fluxional groups could enhance enantioselectivity, providing a scalable method for the synthesis of enantiopure axial building blocks.

**Scheme 5:**
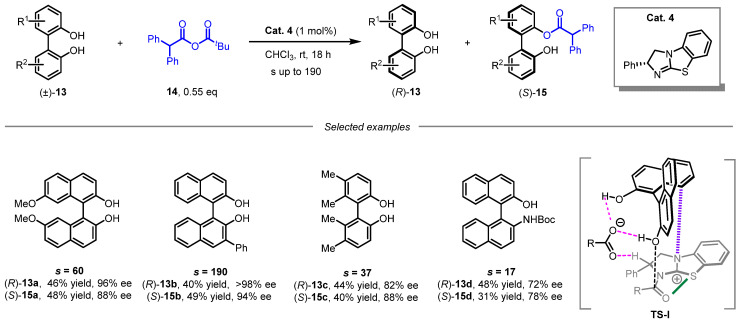
Isothiourea-catalyzed regioselective acylative kinetic resolution of axially chiral biaryl diols.

In 2019, the Smith group reported an efficient catalytic system for the kinetic resolution of unprotected 1,1′-biaryl-2,2′-diols (Scheme 5) [48]. Utilizing isothiourea organocatalyst **4**, in conjunction with a bench-stable mixed anhydride **14**, the team effectively leveraged the Lewis basicity of isothiourea to activate the acyl donor. Employing 2,2-diphenylacetic pivalic anhydride as the optimal acylating agent minimized diacylation while enhancing selectivity. This method operated with a low catalyst loading of just 1 mol% under straightforward reaction conditions, showcasing its high practicality.

**Scheme 6:**
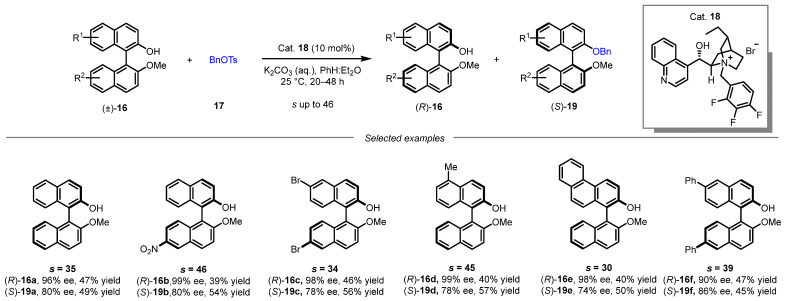
Chiral counterion-mediated practical kinetic resolution of BINOLs.

The approach was applicable to a variety of structures, including symmetric binaphthyl **13a**, biphenyl **13c**, and protected NOBIN derivative **13d**. Remarkably, it could be efficiently scaled up to gram quantities without sacrificing enantioselectivity. Furthermore, this method even facilitated the regioselective KR of unsymmetrical diol **13b** bearing a single 3-substituent, achieving selectivity factors up to 190 and enantiomeric ratios of the recovered unreacted diols exceeding 99:1. Mechanistic investigations revealed the crucial role of the two hydroxyl groups on the diols, which stabilized the transition state through hydrogen bonding (Scheme 5, TS-I). The isothiourea catalyst **4** differentiated each enantiomer of BINOL, while benefiting from the electrostatic stabilization provided by the biaryl’s π-system. The difficulty in acylation of 3,3′-disubstituted BINOL derivatives stemmed from the fact that a 3/3′-substituent introduced an unfavorable steric contact with the acyl group of the acylated catalyst. This result was applied to the highly regioselective acylative kinetic resolution of unsymmetrical biaryl diol substrates bearing a single 3-substituent. In 2017, the Smith group successfully constructed a series of chiral BINOL derivatives through a dynamic kinetic *O*-alkylation of racemic 2-tetralones using a cationic catalyst derived from the cinchona alkaloid [49]. As a continuation of their research, they developed a strategy employing similar catalysts, with benzyl tosylate serving as the alkylating agent, for the kinetic resolution of racemic BINOLs **16** (Scheme 6) [50]. With a cinchona-derived quaternary ammonium salt **18**, the reaction proceeded in a benzene/ether solvent system with aqueous K_2_CO_3_ as the base, enabling a high selectivity in differentiating BINOL enantiomers. The system achieved selectivity factors up to 46, with recovered unreacted BINOLs showing an enantiomeric excess as high as 99% ee.

In addition, their approach had a broad substrate scope, applicable to both *C*_2_-symmetric and non-*C*_2_-symmetric BINOLs (e.g., monomethyl BINOL, 6′-nitro BINOL). It tolerated various substituents at different positions on the binaphthyl framework. However, the *C*_2_-symmetric monomethyl BINOLs bearing a substitution in the 4,4′ or 5,5′ position were relatively sensitive to the size of the substituents: 4,4′-diphenyl or 5,5′-diphenyl substrates exhibited significantly less selectivity compared to bromine substituents at the same position. The reaction could be conducted at room temperature and scaled up to multi-gram quantities (22 mmol, 10.1 g) without sacrificing enantioselectivity, yielding unreacted BINOL **16c** at 43% with 98% ee.

**Scheme 7:**
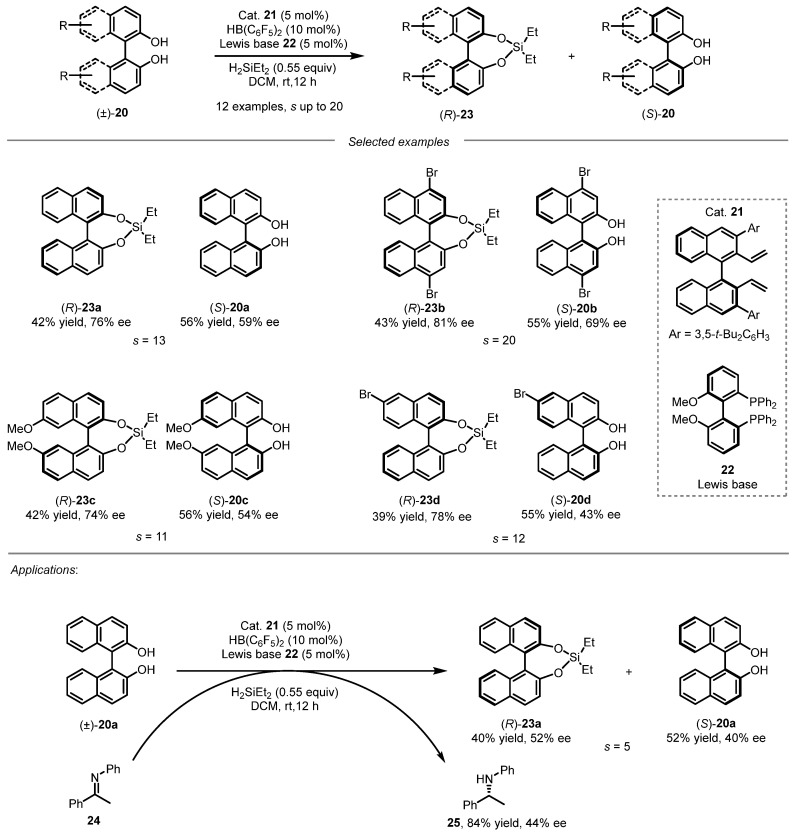
Kinetic resolution of biaryl diols via dehydrogenative coupling with hydrosilanes catalyzed by chiral FLPs.

Recently, the Du group effectively mediated the kinetic resolution dehydrogenative coupling reaction between racemic unprotected BINOLs **20** and hydrosilane by utilizing their developed chiral frustrated Lewis pair (FLP) catalysts with axially chiral olefin skeletons (Scheme 7) [51]. Under optimized conditions, this catalytic system demonstrated an impressive substrate applicability, tolerating BINOLs with substituents at the 3-, 4-, 5-, and 6-positions of the binaphthyl framework, along with various biphenol derivatives. The resulting chiral siloxanes were isolated in yields ranging from 21% to 46% with up to 81% ee, while enantioenriched BINOLs and biphenols were recovered in high yields (up to 69% ee). The selectivity factors ranged from 2 to 20, indicating that the steric hindrance and electronic properties of the biaryl core modulated the stereodiscrimination process. Subsequently, the dehydrogenative coupling reaction was further applied to the hydrogenation of imine. Under the catalysis of Flp, the imine **24** captured H_2_ released during the dehydrogenative coupling of diols and hydrosilanes, yielding secondary amine **25** with yields of 84% and 44% ee. However, the selectivity of the kinetic resolution significantly decreased.

### 2.2. Organocatalytic Kinetic Resolution of Axially Chiral Biaryl Amines

Axially chiral amines, such as BINAM derivatives, are critical for asymmetric catalysis and chiral materials [52], yet efficient catalytic synthesis remains challenging, with traditional approaches heavily reliant on laborious chromatographic separation or stoichiometric resolution protocols. Kinetic resolution, particularly when integrated with organocatalysis, has emerged as a transformative strategy to address this gap, enabling an enantioselective access to enantioenriched BINAM derivatives.

**Scheme 8:**
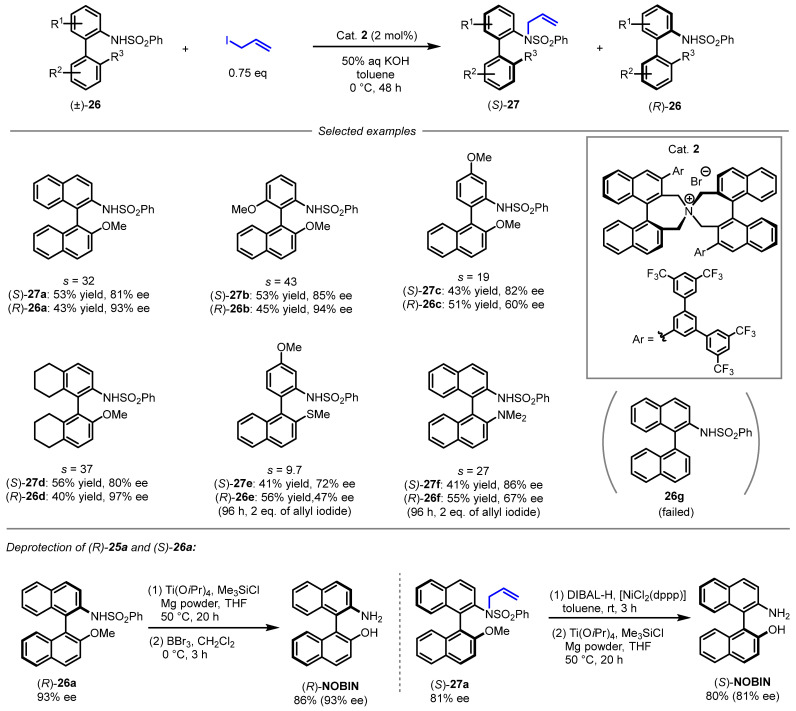
Phase-transfer catalysis-mediated kinetic resolution of axially chiral 2-amino-1,1′-biaryls via N-Allylation.

A significant breakthrough came in 2013 when Maruoka’s team introduced a highly selective kinetic resolution strategy through phase-transfer-catalyzed N-allylation (Scheme 8) [53]. By employing binaphthyl-modified chiral quaternary ammonium salts as catalysts, they optimized conditions using the NOBIN derivative (±)-**26a**, achieving a selectivity factor of 32. The optimized catalyst (*S*, *S*)-Cat.**2**, featuring radially extended aromatic substituents combined with allyl iodide, yielded the allylation product (*S*)-**27a** with an 81% enantiomeric excess (ee) and 53% yield, while recovering (*R*)-**26a** with a 93% ee at a yield of 43%. Notably, lower conversion rates enhanced the product’s ee up to 90%.

This methodology demonstrated a broad substrate scope, effectively resolving biaryl- and biphenyl-structured 2-amino-1,1′-biaryls **26b**–**f** bearing diverse substituents, achieving selectivity factors up to 43. Unfortunately, this method was not applicable to the kinetic resolution of simple 2-amino-1,1′-binaphthyl compound **26g** due to its low reactivity. Importantly, protecting groups can be readily removed, yielding enantiopure NOBIN without a loss of enantiopurity (Scheme 8, bottom). Overall, this breakthrough provided an efficient and selective approach for accessing axially chiral amino compounds, marking a valuable advancement in the catalytic asymmetric synthesis of such scaffolds.

**Scheme 9:**
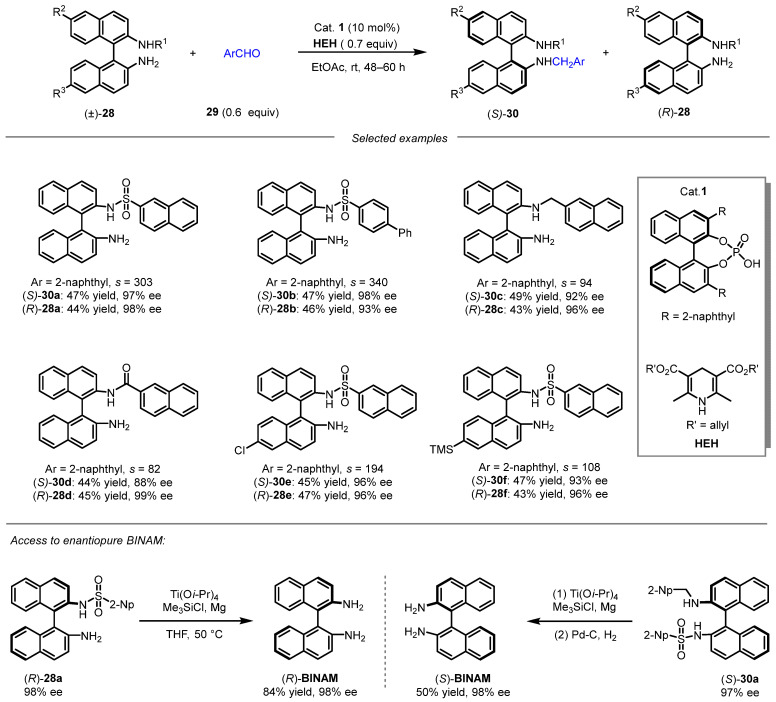
Kinetic resolution of BINAM derivatives via Brønsted acid-catalyzed reductive amination.

During the same period, Tan and co-workers reported an elegant strategy for the kinetic resolution of axially chiral BINAM derivatives by leveraging a chiral Brønsted acid-catalyzed cascade reaction involving imine formation and transfer hydrogenation (Scheme 9) [54]. After optimizing the reaction conditions with model substrate *rac*-**28a**, they identified chiral phosphoric acid Cat.**1**, ethyl acetate as a solvent and Hantzsch ester as a reductive reagent, achieving a remarkable selectivity factor of up to 303. This method accommodated various aldehyde functional groups, protecting groups on the BINAM scaffold, and different substituents, resulting in both products and recovered starting materials with high yields and excellent enantioselectivities (up to 99% ee). For instance, various types of protecting groups such as sulfonyl, benzoyl, 2-naphthylmethyl, fmoc, amido and thioamido groups all demonstrated an excellent compatibility in this reaction (*s* = 7–340). Substrates containing halogens or TMS functional groups at the 6-position or 6,6′-positions of the BINAM framework were also effectively reacted in this reaction and achieved good yields and an excellent enantioselectivity (*s* = 11–307). As a result, the crosscoupling reactions of halogen-containing products also provided opportunities for the efficient construction of more complex BINAM derivatives. Upon the removal of protecting groups from the products, enantiopure BINAM could be obtained, and a single recrystallization yielded products with 100% ee. Their work filled the gap in the catalytic kinetic resolution of axially chiral BINAM derivatives, offering a general approach for constructing enantioenriched BINAM-based compounds, and paved the way for resolving similar axially chiral molecules.

**Scheme 10:**
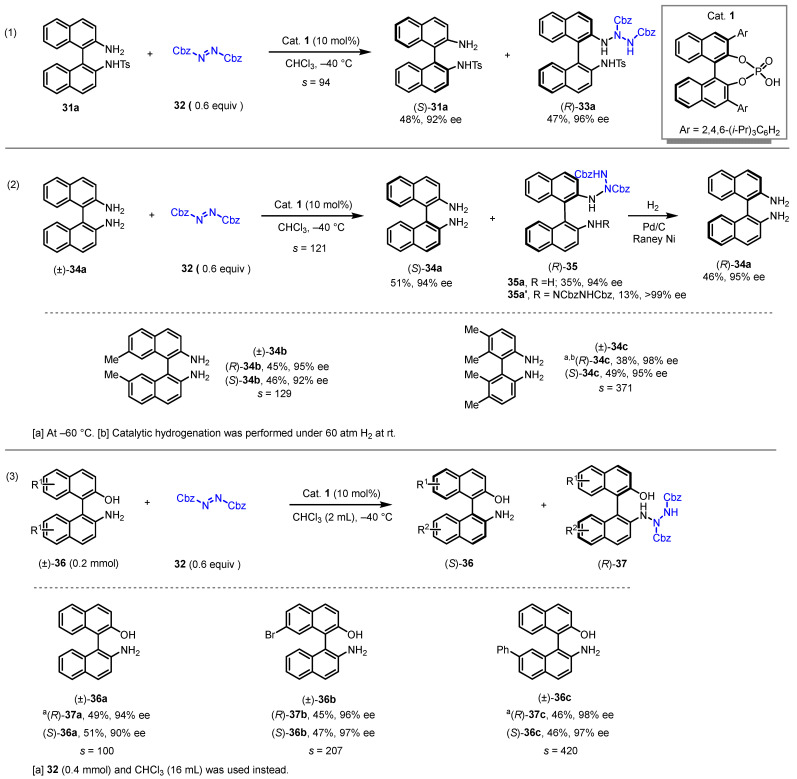
Kinetic resolution of protecting-group-free BINAMs and NOBINs via CPA-catalyzed triazane formation.

Despite these advancements, the existing kinetic resolution methods for BINAMs and NOBINs are often limited by the necessity for protecting groups, which adds extra synthetic steps. To overcome these challenges, the Yang group developed a new methodology for the kinetic resolution of protecting-group-free BINAMs **34** and NOBINs **36** (Scheme 10) [55]. Employing chiral phosphoric acid (CPA)-catalyzed triazane formation with azodicarboxylates, they achieved notable results under optimized conditions using TRIP (Cat.**1**, 10 mol%) as the catalyst. The triazane product **33a** was obtained with a 96% ee, while the recovered (*S*)-**31a** exhibited a 92% ee, resulting in a selectivity factor of 94.

The substrate scope for this method was broad, successfully resolving various mono-N-protected BINAMs with different protecting groups (e.g., Bz, Boc, Cbz, Bn) and achieving high enantioselectivities, with *s*-factors reaching up to 354. Importantly, it was effective for protecting-group-free BINAM **34a**, with an *s*-factor up to 121. It is noteworthy that, during this kinetic resolution process, the triazole product **35a** and a small amount of the bis-triazole product **35a’** were simultaneously formed. Therefore, the authors performed a simple workup procedure, allowing the mixed products **35a** and **35a’** to directly undergo catalytic hydrogenation to ultimately obtain the other enantiomer of BINAM (*R*)-**34a** at a 46% yield with 95% ee. Control experiments indicated that non-reactive NH_2_, NHR, and OH groups in the substrates significantly influenced reactivity and stereoselectivity, likely through hydrogen-bonding interactions with the catalyst.

**Scheme 11:**
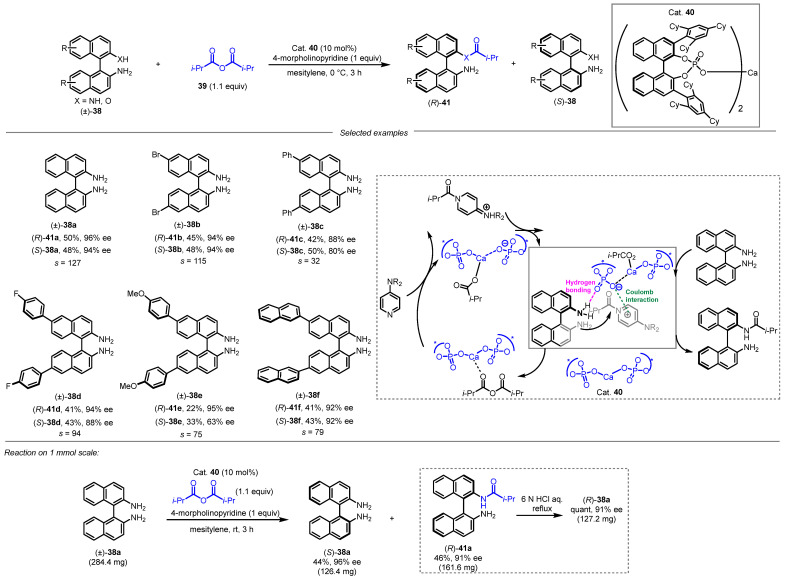
Kinetic resolution of BINAM derivatives via chiral calcium phosphate-catalyzed acylation.

The kinetic resolution of protective-group-free binaphthyl diamines has significant practical value and continues to attract research attention. In 2024, Akiyama reported a chiral calcium phosphate-catalyzed KR strategy for BINAM derivative **38** via acylation with isobutyric anhydride **39** (Scheme 11) [56]. Under optimized conditions, the method could achieve selectivity factors up to 127, with recovered BINAM **38a** and mono-acylated product **41a** exhibiting up to 96% ee. The scope included 6,6′-substituted BINAMs (e.g., bromo, aryl groups), though sterically hindered derivatives (e.g., mesityl) showed a reduced efficiency. A 1 mmol scale reaction proceeded smoothly, and the mono-acylated product could be hydrolyzed to enantiopure BINAM, demonstrating practical utility. Mechanistic insights suggested that the dual hydrogen bonding between the catalyst and BINAM’s amino groups, combined with Coulombic interactions between the chiral phosphate and acylpyridinium intermediate, drove stereoselectivity.

**Scheme 12:**
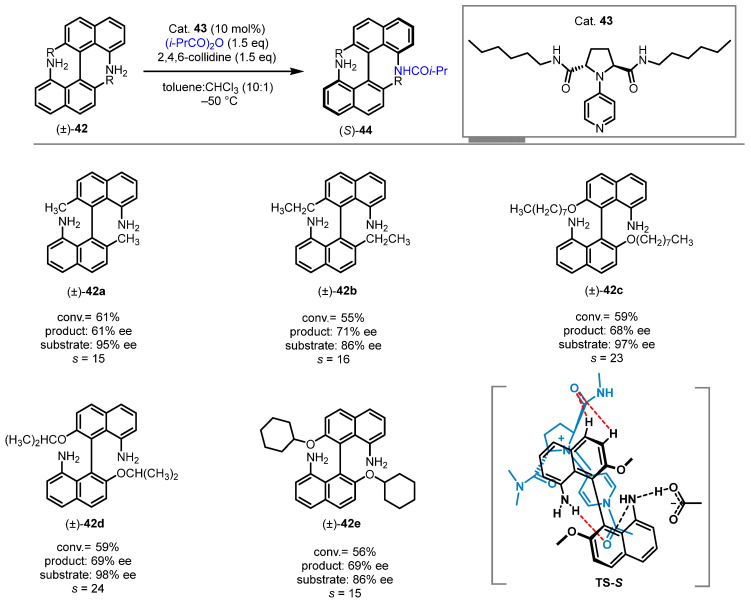
Acylative kinetic resolution of 1,1′-binaphthyl-8,8′-diamines by chiralpyrrolidinopyridine organocatalysts.

Interestingly, OKR emerged as a powerful strategy for accessing a variety of axially chiral amino compounds, including previously unattainable axially chiral diamines. For example, Kawabata et al. developed the first catalytic acylative kinetic resolution of racemic 2,2′-disubstituted 1,1′-binaphthyl-8,8′-diamines using chiral pyrrolidinopyridine organocatalysts (Scheme 12) [57]. The Cat.**43** was able to modulate selectivity, achieving *s*-factors of 15–24 for various derivatives. They found that the presence of an unreacted NH_2_ group was crucial for achieving enantioselectivity, as N,N-dimethyl analogs, which lacked this functionality, displayed a negligible selectivity factor (*s* = 1.1). This result emphasized the significant role of hydrogen bonding in chiral recognition. Moreover, this work established molecular recognition motifs—hydrogen bonding and aromatic stacking—as key design principles for the OKR of challenging amine substrates.

### 2.3. Organocatalytic Kinetic Resolution of Axially Chiral Heteroaryls

Axially chiral heterocyclic compounds, featuring heteroatom-containing backbones, exhibit distinct physical, chemical, and biological properties compared to their all-carbon counterparts. In recent years, the potential applications of these compounds in asymmetric catalysis, pharmaceutical research, and materials science have attracted significant attention from the scientific community [58,59]. However, the inherent structural diversity and unique reactivity of heterocycles still pose challenges for developing universal synthetic methodologies.

**Scheme 13:**
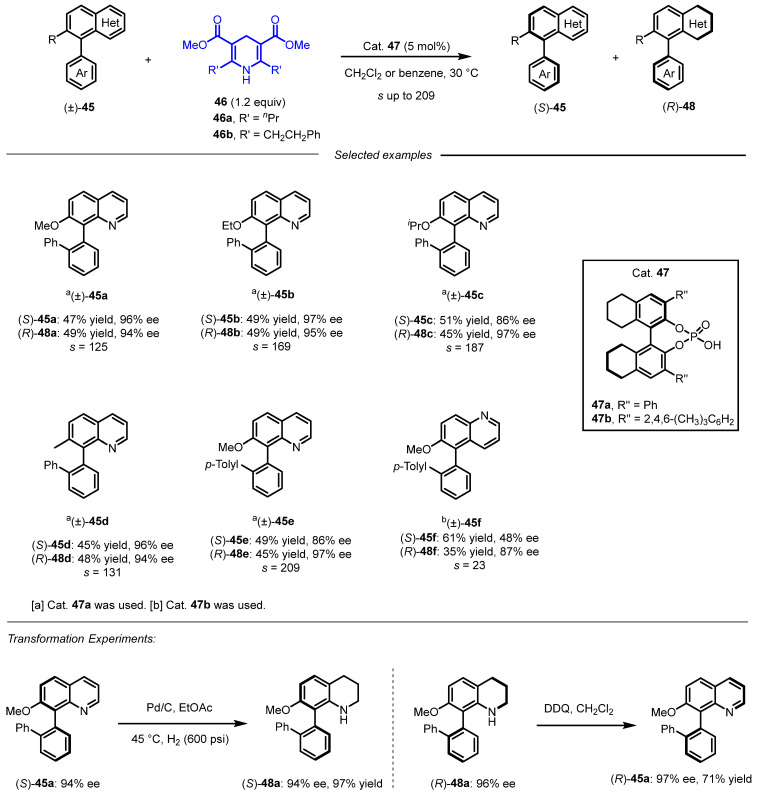
CPA-catalyzed kinetic resolution of axially chiral quinolines via asymmetric transfer hydrogenation.

In 2016, Zhou and co-workers reported the first successful integration of heteroaromatic asymmetric transfer hydrogenation into the KR of axially chiral biaryls, focusing on 5- or 8-substituted quinoline derivatives (Scheme 13) [60]. Utilizing a chiral phosphoric acid (CPA) in conjunction with a Hantzsch ester catalytic system, this method achieved an unprecedented resolution efficiency, allowing the simultaneous generation of two distinct enantioenriched axially chiral skeletons—quinoline and tetrahydroquinoline.

In Zhou’s study, the CPA **47** served a multifaceted role, acting as a hydrogen bond donor to activate the electron-rich quinoline heteroaromatic ring, while also functioning as a chiral selector to facilitate the discrimination between the two enantiomers of the axially chiral biaryl substrates. Initial investigations with iridium-based catalysts fell short of meaningful stereodiscrimination, prompting a shift to the organocatalytic CPA/Hantzsch ester system, which effectively addressed these challenges with a remarkable selectivity factor of 209. The recovered unreacted quinoline substrates and tetrahydroquinoline products displayed enantiomeric excess greater than 94%.

Additionally, the method’s value extended beyond its high selectivity, bridging laboratory research with potential industrial applications. The ability to hydrogenate unreacted quinoline substrate (*S*)-**45a** into tetrahydroquinoline (*S*)-**48a** (via Pd/C catalysis) and to reoxidize tetrahydroquinoline product (*R*)-**48a** back into quinoline (*R*)-**45a** (via DDQ) facilitated a reciprocal conversion. This innovative feature allowed access to both enantiomers of two distinct axially chiral skeletons from a single racemic starting material, exemplifying the transformative potential of the approach in asymmetric synthesis.

**Scheme 14:**
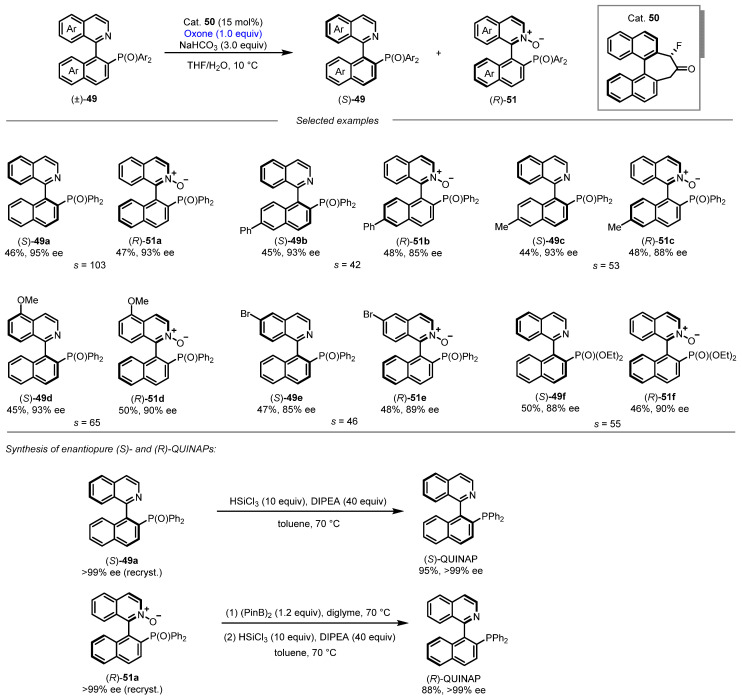
Ketone-catalyzed enantioselective N-oxidation.

QUINAPs (1-(2-diphenylphosphino-1-naphthyl)isoquinolines) emerged as another pivotal class of axially chiral nitrogen-containing compounds with remarkable features in the stereoinduction of diverse enantioselective transformations [61,62]. However, the confined substrate range and extravagant price still posed challenges, limiting their broader utilization. In 2023, the Tan group established an enantioselective kinetic resolution method for the synthesis of axially chiral QUINAPOs by utilizing ketone catalysis (Scheme 14) [63]. This achievement marked the first time that a chiral ketone, Cat.**50**, had been used for the enantioselective oxidation of N atoms. This approach demonstrated an excellent compatibility with various functional groups. For instance, modulating the electronic nature of an aryl moiety with electron-withdrawing, neutral, and donating substituents was well tolerated, affording the corresponding *N*-oxides **51** with up to 93% ee and the recovered QUINAPOs **49** with up to 95% ee. Furthermore, the electronic property of substituents on the isoquinoline moiety had little impact on the enantiocontrol, and the kinetic resolution proceeded smoothly to give the oxidized products as well as the intact substrates with 85–95% ee values. Additionally, variations in the substituent on the phosphorous atom were also attempted; an alkyl or alkoxy group was compatible for this process to give the desired product and the unreacted substrate with the expected efficiency and stereocontrol.

**Scheme 15:**
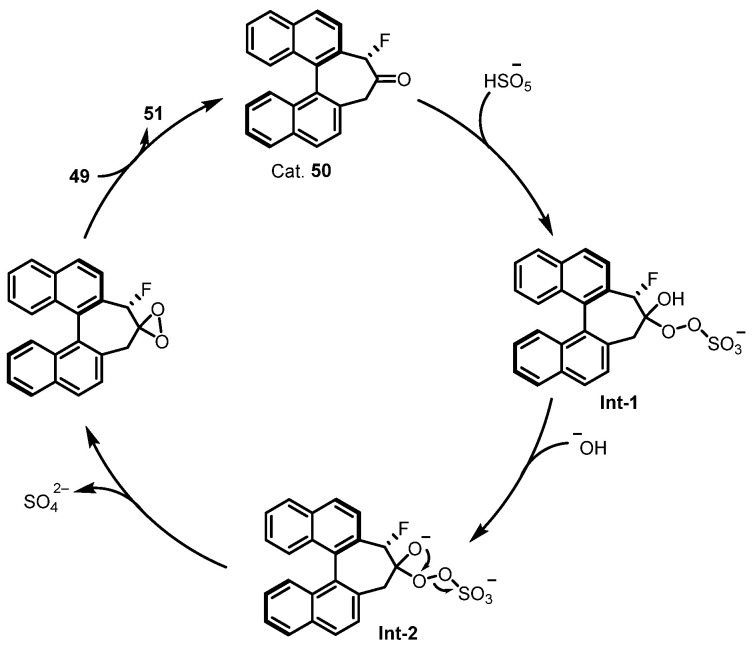
The reaction pathway.

Importantly, the enantioenriched products could be readily converted into the QUINAP targets (Scheme 14, below). Starting from the remaining QUINAPO (*S*)-**41a**, a reduction with HSiCl_3_ could give (*S*)-QUINAP at a 95% yield without the loss of stereochemical integrity. Enantiopure (*R*)-QUINAP was also accessible from the oxidized (*R*)-**42a** through the successive reduction of *N*-oxide and *P*-oxide at a high efficiency. Mechanistic investigations indicated that a dioxirane, generated through the oxidation of the ketone with oxone, acted as the active catalytic species (Scheme 15). Furthermore, the authors had successfully extended this catalytic system to the kinetic resolution of QUINOLs and the dynamic kinetic transformation of pyridine analogs of QUINAPO possessing a labile stereogenic axis. The practicality of the developed protocol was further demonstrated through the successful application of QUINAPO *N*-oxide as a Lewis base catalyst in a series of enantioselective transformations.

**Scheme 16:**
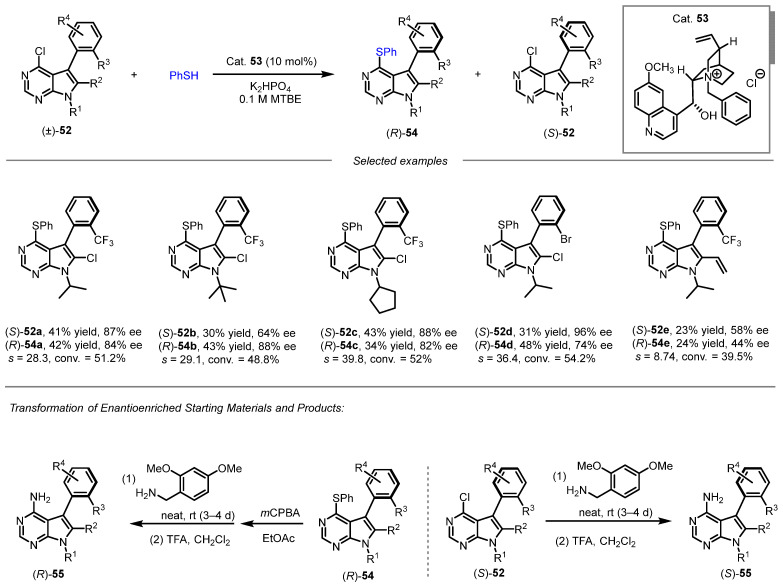
Enantioselective synthesis of pyrrolopyrimidine scaffolds through chiral ammonium salt directed S_N_Ar.

In 2015, the Gustafson group demonstrated that the strategic rigidification of pyrrolopyrimidine-based kinase inhibitors (PPYs) through the control of heterocyclic atropisomerism could serve as an effective strategy for enhancing kinase selectivity [64]. Building on this foundation, the team subsequently developed a quaternary ammonium salt-catalyzed SNAr for the kinetic resolution of 3-aryl PPYs **52** (Scheme 16) [65]. By using thiophenol as the nucleophile, this method achieved selectivity factors of up to 58. Both enantiomerically enriched starting materials and S_N_Ar products underwent stereodivergent functionalizations—such as oxidation to sulfones and amination—without racemization. The S_N_Ar mechanism was shown to operate through a chiral induction by quinine-derived catalysts, with hydrogen bonding and steric repulsion collaboratively directing nucleophilic attack.

Notably, the *Ra* atropisomer of a 3-aryl PPY derivative exhibited sub-nanomolar inhibition (IC_50_ = 6 nM) against breast tumor kinase, with an over 225-fold selectivity compared to its *Sa* counterpart, underscoring the critical influence of axial chirality on biological activity. The high rotational barriers (≥27.9 kcal/mol) in these PPYs ensured excellent stereochemical stability, which was essential for pharmaceutical applications.

**Scheme 17:**
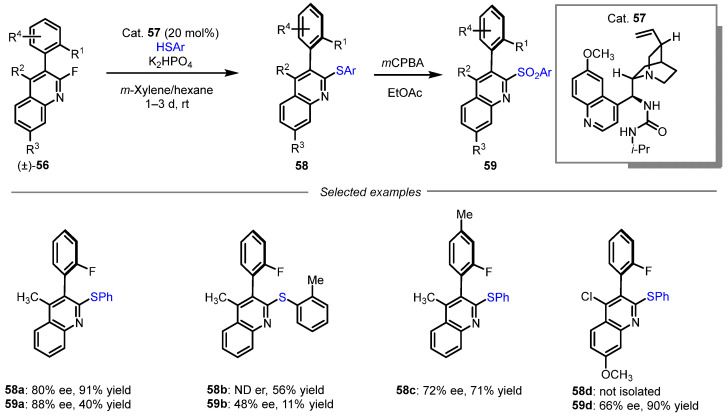
Cinchona alkaloid urea-catalyzed kinetic resolutions for atroposelective 3-arylquinolines via S_N_Ar.

While atropisomerism is increasingly critical in medicinal chemistry, synthetic methods for atropisomeric heterocycles—particularly quinolines—remain underdeveloped compared to biaryl systems. Addressing this gap, Gustafson and colleagues further developed a catalytic atroposelective synthesis of pharmaceutically relevant 3-arylquinolines. Their method employed Cinchona alkaloid-derived ureas to mediate the nucleophilic aromatic substitution of thiophenols with 3-aryl-2-fluoroquinolines (Scheme 17) [66]. It is worth noting that, in some cases, mCPBA was needed to oxidize the products sulphides **58** to sulphones **59** in order to separate the products from the starting material and evaluate the enantioselectivities. A key mechanistic insight was the observation of a reaction continuum between dynamic kinetic resolution and kinetic resolution, which was governed by the substrate’s stereochemical stability: low-barrier substrates favored a DKR pathway, high-barrier substrates followed KR, and those with intermediate barriers exhibited hybrid characteristics.

**Scheme 18:**
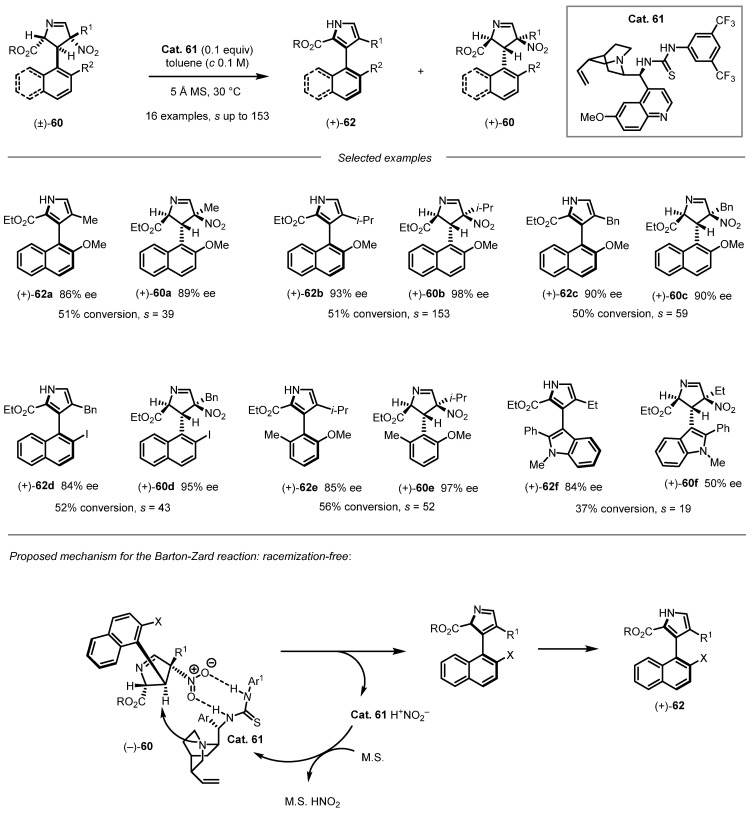
Kinetic resolution of (±)-3,4-dihydro-2H-pyrroles through enantioselective aromatization.

In parallel, axially chiral five-membered heterocycles such as 3-arylpyrroles have remained challenging targets due to the inherent difficulty in controlling axial chirality within small heterocyclic systems. A breakthrough came in 2019 when the Zhu group reported a transformative kinetic resolution strategy via the enantioselective aromatization of racemic 3,4-dihydro-2H-pyrroles—elusive intermediates in the Barton–Zard reaction—using a quinine-derived thiourea catalyst (Scheme 18) [67]. The process began with racemic dihydropyrroles synthesized as single diastereomers via nitroalkene–isocyanoacetate condensation. Chiral thiourea catalyst **61** then enabled kinetic resolution through an elimination with remarkable selectivity (*s*-factors up to 153), affording both recovered dihydropyrroles **60** (up to 98% ee) and pyrrole products **62** (up to 93% ee). The system accommodated diverse alkyl, alkoxycarbonyl, and aryl substituents and demonstrated a gram-scale applicability.

Mechanistic studies indicated that the catalyst promoted a syn elimination of HNO_2_, bypassing racemization-prone 3H-pyrrole intermediates. Bifunctional hydrogen bonding and tertiary amine activation enabled stereoselectivity, acting as an alternative to the classic Barton–Zard mechanism (Scheme 18, below). This study advanced the synthesis of axially chiral molecules by integrating kinetic resolution with mechanistically innovative aromatization, offering a scalable and enantioselective platform for heterobiaryl atropisomers.

### 2.4. Organocatalytic Kinetic Resolution of Biaryl Compounds with Axial and Central Chirality

Traditional methodologies have largely concentrated on the synthesis of axially chiral compounds as isolated entities. However, the challenge escalates when aiming to incorporate multiple stereogenic centers, specifically both axial and central chirality, within a single framework. This complexity necessitates innovative strategies to streamline their synthesis. Organocatalytic kinetic resolution offers a promising solution by enabling the concurrent generation of central chirality alongside the resolution of axial chirality. This dual capability allows for the efficient construction of diverse chiral architectures in a single synthetic step, presenting significant implications for asymmetric synthesis and the development of complex chiral molecules.

**Scheme 19:**
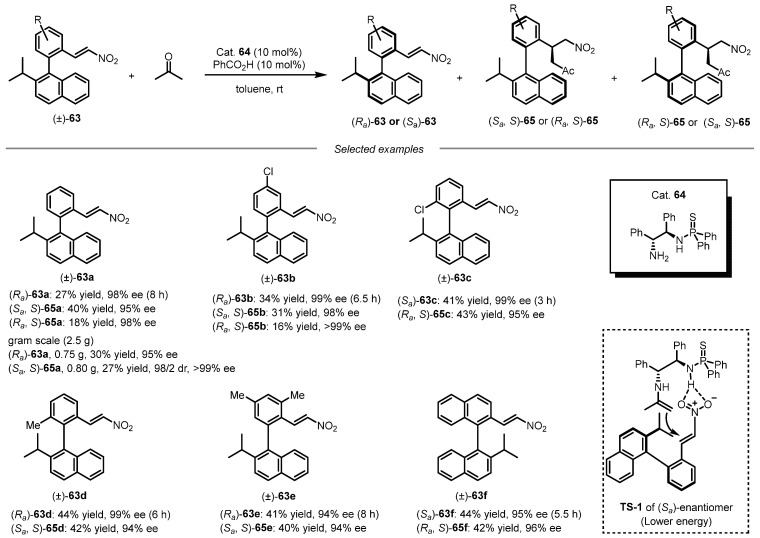
Kinetic resolution of 2-nitrovinyl biaryls catalyzed by a bifunctional thiophosphinamide.

**Scheme 20:**
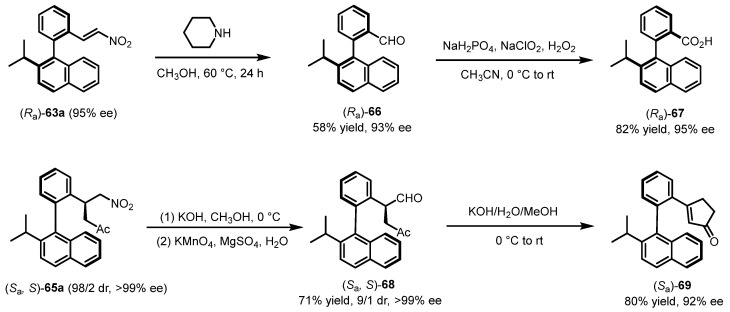
Transformation experiments.

An efficient kinetic resolution of axially chiral 2-nitrovinyl biaryls has been illustrated by our group (Scheme 19) [68]. We first applied the asymmetric Michael addition of nitroolefins to the kinetic resolution of axially chiral biaryls bearing olefinic functionality with high levels of enantioselectivity. The ability of primary amine/thiophosphinamide **64** to distinguish the chiral axis of biaryls to provide an efficient kinetic resolution was achieved [69,70,71,72]. Racemic 2-nitrovinyl biaryls **63** were converted into highly enantiomer-enriched 2-nitrovinyl biaryls at 20–44% yields with ee values ranging from 90 to 99% and a pair of separable Michael addition products **65** bearing both axial and central chiralities with excellent stereocontrol. Moreover, axially chiral nitroolefins with a fused ring system were resolved with a similarly high selectivity, all substrates examined were recovered at 93–>99% ee with 27–44% isolated yields. Notably, there was a substantial rise in the kinetic resolution selectivity when methyl isobutyl ketone was employed as the rection partner instead of acetone. The reaction could also be easily scaled up. A test reaction with *rac*-**63a** worked similarly well at a gram scale to yield enantiomerically enriched (a*R*)-**63a** (95% ee) at a 30% yield and the major diastereomer (*S_a_*, *S*)-**65a** (98/2 dr, >99% ee) at a 27% yield after a single recrystallization. Moreover, both the recovered 2-nitrovinyl biaryls and the Michael addition products could be further transformed into a plethora of novel axially chiral biaryls bearing diverse functionalities (Scheme 20).

The hypothetical stereochemical model was shown in the transition state (TS-1) of the (*S_a_*)-enantiomer; the bulky isopropyl group and the enamine moiety were located far apart, and there was no steric repulsion between them to destabilize the transition state. However, in the transition state of the (*R_a_*)-enantiomer, there existed a serious van der Waals repulsion between the bulky isopropyl group and the enamine moiety. Therefore, the activation energy for the reaction of the (*S_a_*)-enantiomer was considerably lower than that of the (*R_a_*)-enantiomer, which became the major recovered isomer because it reacted slower.

**Scheme 21:**
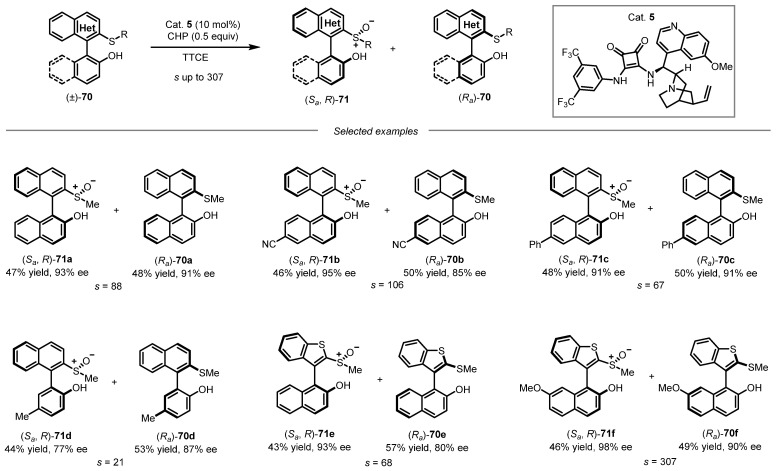
Bifunctional squaramide-catalyzed oxidative kinetic resolution.

Axially chiral thioethers and sulfoxides play a pivotal role in asymmetric transformations, owing to their unique stereodifferentiating capabilities. However, their practical applications are severely constrained by the absence of efficient, scalable methodologies for accessing structurally diverse enantiopure derivatives. In 2024, the Guo group reported a novel oxidative kinetic resolution using a chiral bifunctional squaramide catalyst and cumene hydroperoxide (CHP) (Scheme 21) [73]. For the first time, the cinchona-derived squaramide catalyst enabled enantioselective sulfur oxidation in 1,1,2,2-tetrachloroethane (TTCE), achieving up to 93% ee and >20:1 dr for sulfoxides with dual axial/S-central chirality, and an s-factor up to 307.

The method tolerated diverse biaryl/heteroaryl (e.g., benzothiophene) thioethers, including electron-donating/withdrawing and steric substituents. For example, the authors successively examined biaryl thioethers **70** with substitutions at the C8 and C7 positions, or with different substituents at the C6 position (including cyano, formyl, ester, phenyl and substituted phenyl groups). Under the optimal conditions, all of these were well tolerated, thereby providing the corresponding (*S_a_*, *R*)-**71** and the recovered (*R_a_*)-**70**, both with high enantioselectivities.

Gram-scale reactions and ligand derivatization validated the practical utility. This work expanded chiral squaramide catalysis to sulfur oxidation, offering a scalable paradigm for multi-chiral heteroatomic compounds.

Mechanistic investigations revealed that hydrogen bonding was the key to selectivity control. The squaramide moiety of the catalyst activated CHP via dual hydrogen bonding, while the tertiary amine group interacted with the phenolic hydroxyl group of the substrate, orienting the thioether moiety for a stereoselective attack on the activated CHP. These synergistic non-covalent interactions created a precise chiral microenvironment, ensuring high stereocontrol efficiency.

## 3. Organocatalytic Kinetic Resolution of C-N Axially Chiral Molecules

C-N axial chirality, characterized by a stereogenic axis formed between carbon and nitrogen atoms, presents distinct synthetic challenges. The C-N bond generally exhibits lower rotational barriers than the C-C bond, which can compromise configurational stability and necessitate a precise stereochemical control during synthesis. Moreover, the structural diversity and unique reactivity of C-N axially chiral compounds have impeded the development of universal synthetic methodologies. Despite these inherent difficulties, remarkable progress has been achieved in recent years [74,75,76,77], as exemplified by the successful ORK strategy.

**Scheme 22:**
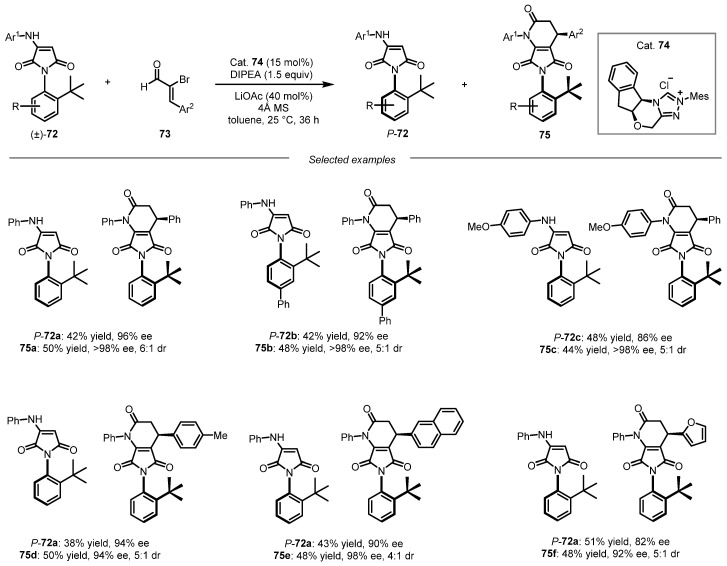
Chiral NHC-catalyzed kinetic resolution of *N*-aryl aminomaleimides.

One notable advancement was the novel chiral *N*-heterocyclic carbene (NHC)-catalyzed kinetic resolution strategy developed for racemic *N*-aryl aminomaleimides reported by the Biju group, enabling access to enantioenriched C−N axially chiral derivatives via remote chirality control (Scheme 22) [78]. The method leveraged chiral triazolium salt-derived NHCs to generate *α*,*β*-unsaturated acylazolium intermediates from 2-bromoenals **73**, which selectively underwent [3+3] annulation with one enantiomer of *N*-aryl aminomaleimides. Under optimized conditions, the reaction yielded fused dihydropyridinones **75** (bearing both axial and central chirality, >98% ee, up to 6:1 dr) and recovered enantioenriched *N*-aryl aminomaleimides **72** (up to 96% ee). Mechanistic studies confirmed the remote chirality induction via steric interactions between the catalyst’s aminoindanol moiety and the substrate’s *t*-Bu group. Rotational barrier analyses (both experimental and DFT) established the stability of axial chirality, with barriers around 28–30 kcal/mol. These findings highlighted the expanded utility of NHC catalysis in axially chiral synthesis and provided an efficient route to C−N axially chiral compounds, offering new insights into remote stereocontrol mechanisms.

**Scheme 23:**
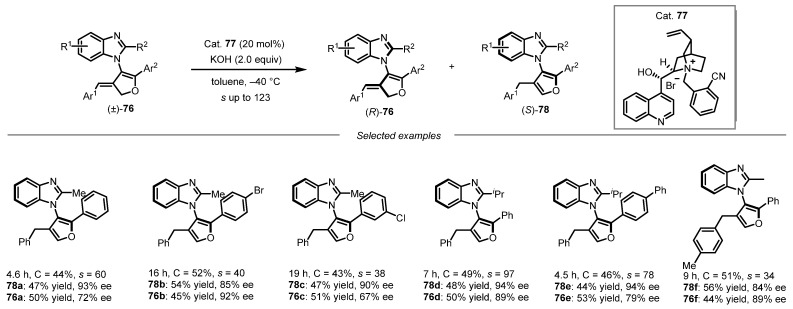
Kinetic resolution of axially chiral furan-benzimidazoles via olefin isomerization.

In another significant development, the Guo group introduced a phase-transfer-catalyzed kinetic resolution strategy for the synthesis of axially chiral methylene dihydrofuran-benzimidazoles (*R*)-**76** (Scheme 23) [79], critical intermediates for furan-benzimidazoles in medicinal chemistry. By employing a chiral ammonium catalyst **77**, racemic substrates **76** underwent intramolecular [1,3]-H transfer to yield enantioenriched furan-benzimidazoles **78** (up to 96% ee) alongside recovered methylene dihydrofuran precursors **76** (up to 96% ee), achieving selectivity factors up to 123. By combining KR with stereospecific isomerization, the method offered a versatile platform for accessing structurally diverse axially chiral furans and benzimidazoles, with implications for catalysts design and bioactive molecule discovery.

**Scheme 24:**
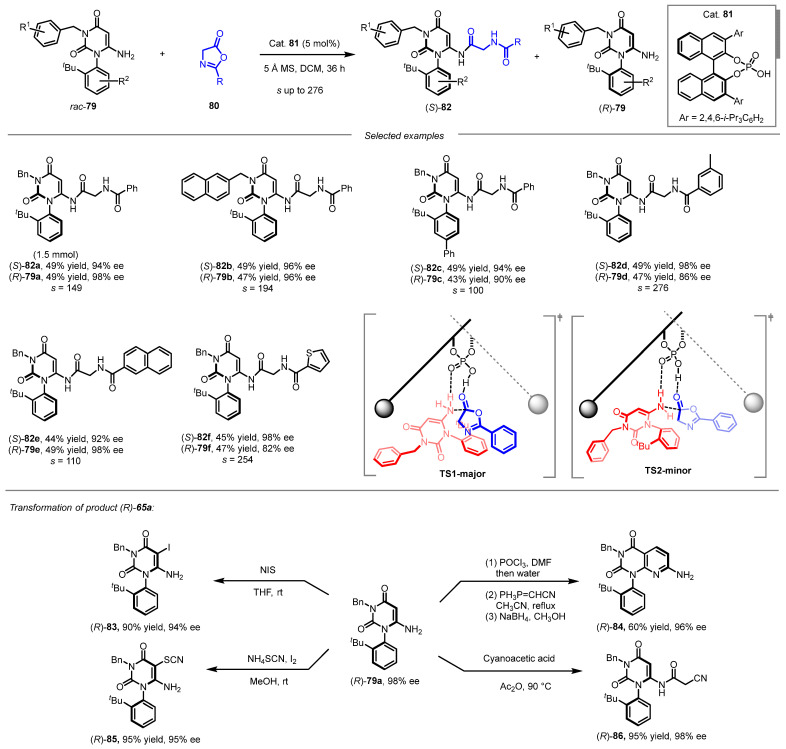
CPA-catalyzed asymmetric synthesis of axially chiral uracils via kinetic resolution.

The asymmetric synthesis of C–N axially chiral uracils, important in the development of pharmaceuticals such as PDE4 inhibitors and KRAS G12C antagonists, had long been constrained by limited catalytic methods, historically relying on chiral chromatography or auxiliary strategies. In 2025, Li and colleagues reported a groundbreaking chiral phosphoric acid (CPA)-catalyzed kinetic resolution of 6-NH_2_-substituted uracils using achiral azlactones as acylating reagents, thus enabling an efficient access to enantioenriched uracils and their acylated derivatives (Scheme 24) [80].

Using Cat.**81**, this method achieved selectivity factors up to 276, yielding acylated uracils with an excellent enantiomeric excess of up to 98%, alongside recovered uracils also exhibiting up to 99:1 er. The method accommodated a wide range of *N*-aryl/alkyl uracils and azlactones, including various halogenated, heterocyclic, and polycyclic substituents. Notably, large-scale reactions and subsequent derivatizations (such as iodination, pyridine formation, and thiocyanation) validated its practical applicability while preserving a high enantiopurity (Scheme 24, below). Furthermore, to investigate the stereochemical results of the reaction, the authors performed density functional theory calculations. The calculations indicated that TS1 had 1.8 kcal mol^−1^ less energy than TS2, and the predicted ee value was 91%, which closely aligned with the experimentally observed ee value. Therefore, TS1 was likely to be the major transition state.

Overall, the application of OKR in the synthesis of C-N axial chirality offered innovative methodologies that significantly improved the efficiency and versatility of producing structurally diverse axially chiral compounds, with important implications for asymmetric catalysis and drug discovery.

## 4. Organocatalytic Kinetic Resolution of Axially Chiral Styrenes

Axially chiral styrenes are a fascinating subclass of atropisomeric molecules characterized by a restricted rotation around a stereogenic axis linked to a styrenic double bond, typically involving C(sp^2^)–C(aryl) or C(sp^2^)–C(heteroaryl) bonds. The unique axial chirality in these compounds arises from the rigidity and orientation of substituents around the double bond, resulting in two relatively non-interconvertible stereoisomers. However, in contrast to the well-explored biaryl and heterobiaryl atropisomers, axially chiral styrenes often display low rotational barriers due to their relatively small steric hindrance [81,82,83,84]. This characteristic poses significant challenges in achieving stable configurations, complicating their characterization and isolation and making catalytic asymmetric synthesis particularly demanding.

**Scheme 25:**
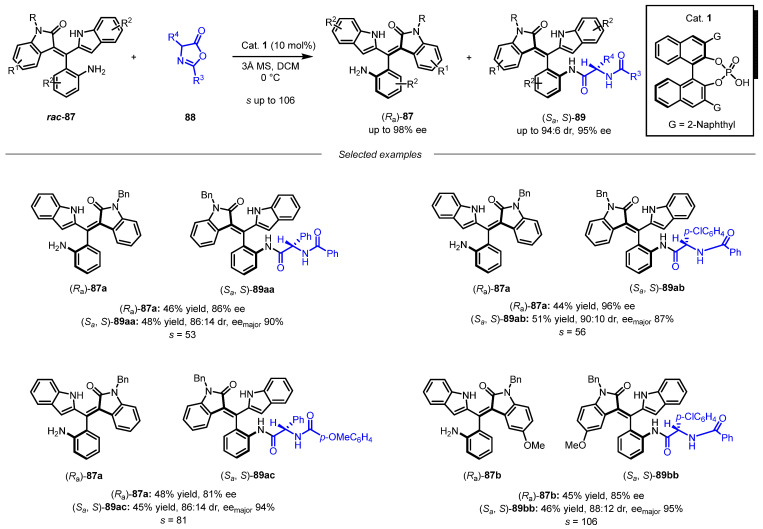
CPA-catalyzed atroposelective synthesis of axially chiral oxindole styrenes via kinetic resolution.

Despite these challenges, considerable progress has been made in the synthesis of axially chiral styrenes. The application of kinetic resolution through organocatalytic strategies has enhanced the efficiency of producing these complex chiral structures and broadened the range of accessible axially chiral styrenes, thereby expanding their potential applications in various fields. A landmark advancement was reported by the Shi group in 2020, which presented the first atroposelective synthesis of oxindole-based axially chiral styrenes via catalytic kinetic resolution. This work effectively bridged a critical gap in accessing this class of atropisomers (Scheme 25) [85]. By utilizing chiral phosphoric acid catalyst **1** in conjunction with azlactones **88** as a resolving reagent, the racemic oxindole styrenes **87** underwent kinetic resolution to yield enantioenriched products (*R_a_*)-**87** with up to 98% enantiomeric excess and selectivity factors reaching as high as 106. The method also enabled the formation of bisamide derivatives **89** exhibiting both axial chirality (up to 94:6 dr) and central chirality (up to 95% ee) through a stereoselective ring-opening of azlactones.

**Scheme 26:**
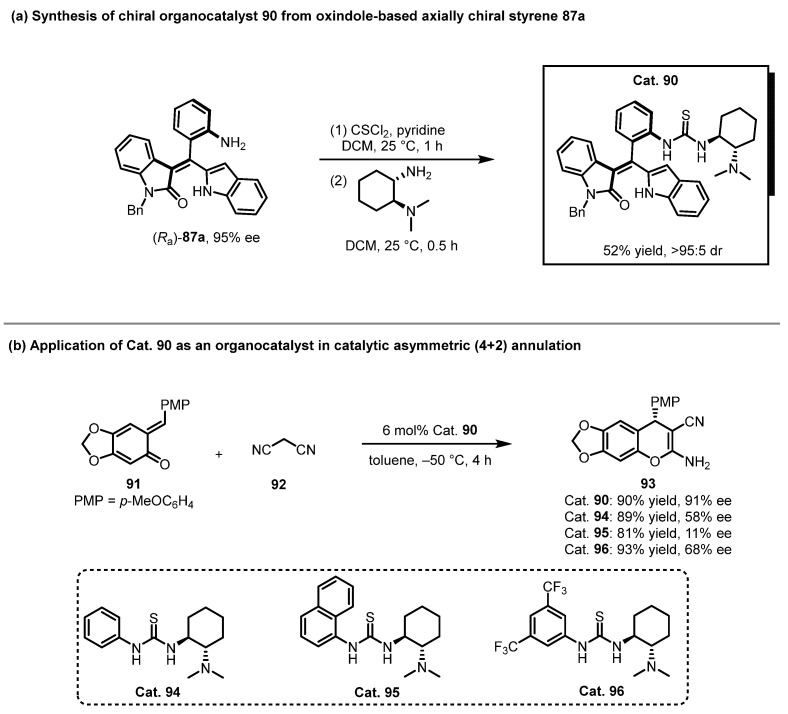
Synthesis of chiral organocatalyst **90** from oxindole-based axially chiral styrene (*Ra*)-**87a** and application in catalytic asymmetric (4+2) annulation.

The research demonstrated an excellent tolerance for diverse substituents on both the oxindole and styrene scaffolds, accommodating various electron-donating and electron-withdrawing groups, as well as heterocyclic systems. Additionally, the scalability of the method to gram-scale synthesis, coupled with successful downstream derivatizations, emphasized its practical utility. Notably, the recovered styrene (*Ra*)-**87a** could be converted into the chiral thiourea-phosphine catalyst **90** that exhibited superior enantiocontrol (up to 91% ee) in asymmetric annulation reactions compared to existing catalysts (Scheme 26).

**Scheme 27:**
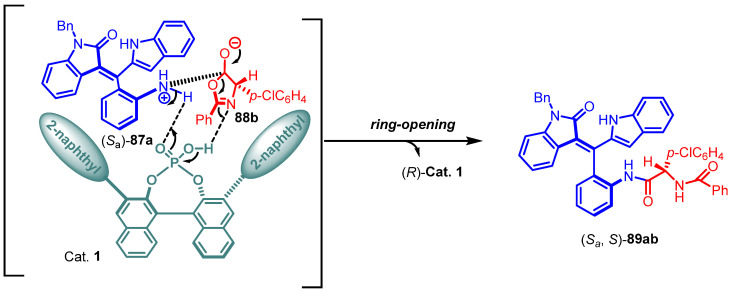
Plausible model to explain the absolute stereochemistry of products **89**.

Mechanistically, the ability of CPA to facilitate enantioselectivity was derived from dual hydrogen bonding interactions with both the substrates and azlactones (Scheme 27). These interactions stabilized transition states and enhanced the differentiation of enantiomers. Rotational barrier measurements (ranging from 27 to 30 kcal/mol) confirmed the sufficient configurational stability needed for synthetic applications. Collectively, this research not only expanded the palette of atropisomeric compounds but also laid a strong foundation for future investigations into the synthesis and applications of axially chiral styrenes, marking a significant contribution to the field of axial chirality in organic synthesis.

By incorporating oxindole-based styrenes into the atropisomeric family, Shi’s study offered a robust method for controlling dual stereogenic elements. The versatility of this approach and its potential for developing new chiral organocatalysts—such as thiourea-phosphines—underscored its considerable implications for asymmetric catalysis and drug discovery, effectively addressing long-standing challenges in the synthesis of axially chiral styrenes.

**Scheme 28:**
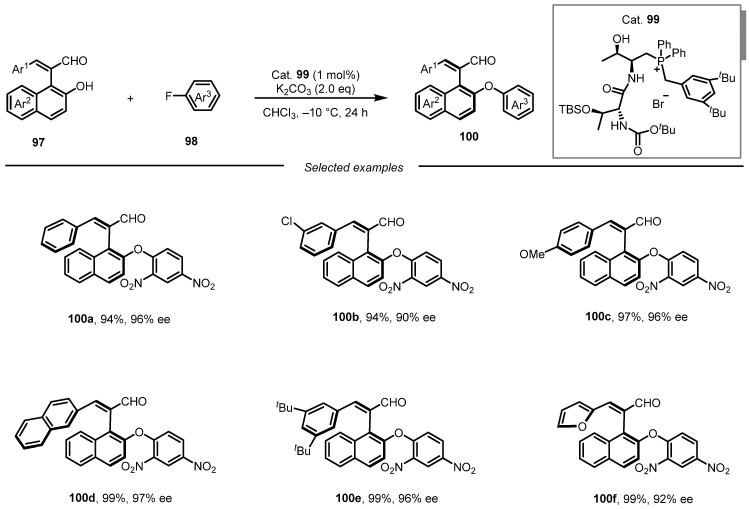
Enantioselective synthesis of axially chiral aldehyde-containing styrenes via S_N_Ar reaction-guided dynamic kinetic resolution.

The dynamic kinetic resolution of racemic starting materials has been one of the most powerful and reliable strategies for the synthesis of enantiopure compounds in past decades. Among them, the dynamic kinetic resolution is also the convenient and efficient route for synthesizing axially chiral styrenes. Atropisomeric carbonyl compounds constitute another type of framework that frequently appears and has received increasing attention in the field of synthesis. In particular, the axially chiral biaryl aldehydes as catalysts play an indispensable role in carbonyl catalysis. However, due to the difficulty in controlling the *E*/*Z* selectivity and the relatively low rotation barrier of axial non-cyclic styrenes compared with the cyclic arylalkenes or the biaryl atropoisomers, the atroposelective synthesis of axially chiral acyclic styrenes remains a formidable challenge. In 2023, the Su and Wang research groups jointly disclosed an asymmetric nucleophilic aromatic substitution reaction of aldehyde-substituted styrenes **97** in the presence of chiral peptide–phosphonium salts **99** involving a dynamic kinetic resolution (Scheme 28) [86]. The S_N_Ar reaction specifically involved treating the aldehyde-containing styrenes bearing (*o*-hydroxyl)aryl unit **97** with common fluoroarenes **98** via an exquisite bridged biaryl lactol intermediate, to obtain a series of axially chiral aldehyde-containing styrenes **100** with different functional groups and bioactive fragments in high stereoselectivities (up to >99% ee) and complete *E*/*Z* selectivities under a very low catalyst loading (1 mol%). During the detailed investigation of the substrate scope, a wide variety of (*o*-hydroxy)aryl-alkene aldehydes installing different substituents on the aromatic rings Ar^1^ and Ar^2^, as well as various nitro-fluorobenzene substrates, were all applicable, and the corresponding axially chiral styrene products were generated in high yields with excellent asymmetric induction. The practicality and effectiveness of this method were also demonstrated through the gram-scale synthesis and facile follow-up reactions, especially in the preparation of novel axially chiral styrene molecules containing an extended C=C unit and carboxylic acid. Furthermore, both experimental and computational studies have shown that the condensation of the phenolic hydroxyl group with the aldehyde group of the racemic styrene substrate to form a hemiacetal intermediate with a five-membered ring was the key step determining the reaction rate and stereoselectivity.

## 5. Conclusions

Over the past decade and a half (2010–2025), organocatalytic kinetic resolution (OKR) has matured from a promising concept into a transformative and indispensable strategy for the asymmetric synthesis of axially chiral compounds. This review has systematically chronicled its successful application across three pivotal scaffolds: C-C biaryls, C-N heterobiaryls, and olefinic systems. By harnessing a diverse arsenal of non-covalent interactions—hydrogen bonding, ion pairing, π-stacking, and electrostatic effects—organocatalysts achieve an exquisite stereodiscrimination, overcoming the inherent challenges of low rotational barriers and limited stereocontrol. The progression from resolving classic BINOLs and BINAMs to accessing intricate heterocyclic QUINAPs, medicinally relevant pyrrolopyrimidines, and stereochemically complex styrenes underscores the remarkable versatility and power of OKR. It provides a sustainable, operationally simple alternative to traditional stoichiometric resolutions and transition metal catalysis, enabling the practical, enantioselective synthesis of privileged chiral building blocks for catalysis, drug discovery, and materials science.

Despite these monumental achievements, the field stands at an inflection point. To transition from demonstrating feasibility to achieving broad utility, researchers must address several key challenges that delineate the frontier for future investigation.

(1) **Expanding the substrate universe**: The current methodologies often excel with specific, privileged scaffolds but struggle with more exotic or functionally dense architectures. Future catalyst design must prioritize broad-spectrum selectivity, capable of resolving substrates with multiple polar functionalities, sterically encumbered environments, or sensitive groups without the need for protective-group manipulations. The successful resolution of protecting-group-free BINAMs and amino alcohols points the way forward.
(2) **Predictive catalyst design and mechanistic elucidation**: While many systems operate with high efficiency, the design of new catalysts for unmet challenges remains largely empirical. A deeper, quantitative understanding of the non-covalent interaction networks governing enantioselectivity is crucial. The integration of advanced computational tools (e.g., DFT, machine learning), coupled with in situ spectroscopic and kinetic studies, will enable the transition from discovery-driven to rational design-driven catalyst development. This will be essential for tackling substrates with minimal steric differentiation.
(3) **Pursuing ideal efficiency**: The fundamental 50% yield limit of classical KR remains a significant practical constraint. The future lies in intelligently merging OKR with DKR and catalytic deracemization protocols. As exemplified by recent work on labile heterobiaryls, designing systems where substrate racemization is efficiently catalyzed under resolution conditions can theoretically deliver the 100% yield of a single enantiomer. Developing mild, organocatalytic racemization pathways compatible with resolution steps is a paramount goal for enhancing synthetic economy.
(4) **Toward application**: The ultimate value of these chiral scaffolds lies in their utility. Future research must bridge the gap between resolution and application by demonstrating gram-to-kilogram scalability under practical conditions (low catalyst loading, benign solvents, simple workups) and focusing on the direct synthesis of application-ready molecules, such as chiral ligands, organocatalysts, or bioactive compound cores, with minimal downstream modification.
(5) **Advancing sustainability**: The inherent “green” credentials of organocatalysis, metal-free and often air- and moisture-tolerant, should be further amplified. Research should aim for catalyst recyclability, the use of biorenewable solvents, and the development of catalytic systems powered by light or other sustainable energy inputs.

In conclusion, organocatalytic kinetic resolution has firmly established itself as a cornerstone of modern asymmetric synthesis for axially chiral compounds. The field now stands at an inflection point, moving beyond proving feasibility to addressing the nuanced challenges of scope, predictability, efficiency, and real-world utility. By focusing on these future perspectives—predictive design, DKR, and scalable functionalization—researchers can unlock the full potential of OKR. This will not only yield novel scientific insights into chirality transfer and molecular recognition but also provide robust, sustainable pipelines for creating complex chiral molecules that will drive innovations in pharmaceuticals, agrochemicals, and advanced materials.

## Data Availability

Not applicable.
